# Psychological, physiological, and biochemical correlations after negative emotional videos in college students with and without premenstrual syndrome

**DOI:** 10.3389/fpsyt.2023.1228276

**Published:** 2023-08-15

**Authors:** Jingyu Xing, Hao Wu, Xue Wang, Shuang Yi, Yu Wei, Yan Zhao, Xingang Hu

**Affiliations:** ^1^School of Chinese Medicine, Beijing University of Chinese Medicine, Beijing, China; ^2^Medical Department, Huguosi Hospital of Beijing University of Chinese Medicine, Beijing, China; ^3^Chemical Industry Press Co., LTD, Beijing, China; ^4^China National Center for Food Safety Risk Assessment, Beijing, China; ^5^Internal Encephalopathy of Traditional Chinese Medicine, Dongfang Hospital of Beijing University of Chinese Medicine, Beijing, China

**Keywords:** PMS, negative emotion regulation, ANS, HPA axis, EEG

## Abstract

**Introduction:**

Women with premenstrual syndrome (PMS) suffer heavily from emotional problems, the pathogenesis of which is believed to be related to the hypothalamic-pituitary-adrenal (HPA) axis, autonomic nervous system (ANS) and central nervous system (CNS). We took into account all 3 aspects to observed the psychological, physiological and biochemical correlations under anger and sadness in college students with and without PMS.

**Methods:**

33 students with PMS and 24 healthy students participated in the emotion induction experiment, and were required to fill out self-report scales. Their salivary cortisol (SCort), skin conductivity level (SCL), heart rate variability (HRV), blood pressure (BP) and electroencephalogram (EEG) data were collected at the resting stage and 10-15 minutes after each video.

**Results:**

Compared to healthy controls, students with PMS showed lower SCort level and higher VLF at rest, and no statistic difference in activities of ANS and HPA axis after emotional videos, but different results in EEG in all conditions. The decreases in SBP during angry video, SCort after angry and neutral videos, and increases in θ band power during sad video were moderately correlated with increases in PMS score. No intergroup differences were found in self-report emotions.

**Discussion:**

Students with PMS had lower activity of HPA axis and possibly higher activity of PNS at rest, and different response patterns in CNS in all conditions. Several EEG frequencies, especially θ band, in specific encephalic regions during emotional videos, as well as declined HPA activities in dealing with angry and neutral stressors, in which γ activity in frontal lobe may play a role, showed moderate correlations with more severe PMS.

## 1. Introduction

Premenstrual syndrome (PMS) is characterized by repeatedly mental, physical and behavioral changes that occur in the luteal phase of women of childbearing age (1). Recent epidemiological studies have shown that the prevalence rate of PMS among female medical college students in China is about 42.8–56.7% (2–4).

PMS often results in myriad distress to social work and interpersonal relationships, which are no less painful than major depressive disorder (5).The suffering is largely on account of abnormal emotional regulation. Studies have found that women with PMS were more sensitive to anxiety (6), endured more negative emotions (7) and stressful events (8, 9), and experienced persistent abnormal emotional states (10). Their attention to negative events are positively correlated with the risk of PMS (11), and the impaired emotional regulation enhanced negative experience, thus exacerbating symptoms (12).

Anger and sadness are noticeable negative emotions among people with PMS. Ding (13) found that the most prominent emotion of PMS group was anger. Saglam et al. (14) found that PMS group showed higher level of anger and lower level of control. Another survey from Sri Lanka pointed out that feeling sad or hopeless was the most frequent reported emotional problem among adolescent females with PMS (15). Our previous research also found that female college students with PMS were more prone to anger and sadness (16). However, few research relative to PMS was conducted on anger and sadness. Gültekin et al. (17) found that, in PMS group, the recognition rate of sad faces in the luteal phase was lower than that in the follicular phase, both of which showed no difference compared with the healthy group. Ma (18) found that in emotional conflict tasks, the PMS group in the luteal phase responded significantly longer to angry faces than to happy faces, and their recognition rate of angry faces in the luteal phase was noticeably lower than that in the follicular phase. Gao et al. (19), through scale evaluation and functional magnetic resonance imaging (FMRI), observed a link between anger disorder and abnormal ReHo levels in the anterior thalamus, superior frontal gyrus, para-central lobule and right cerebellum among women with premenstrual dysphoric disorder (PMDD).

The cause of PMS is not yet conclusive. Research mainly focused on the fluctuations in ovarian hormones, the activities of the central nervous system (CNS) and the autonomic nervous system (ANS), and the adjustment of the hypothalamic–pituitary–adrenal (HPA) axis. Earlier studies suggested that a heightened sensitivity to changes in sex hormone levels is responsible for PMS (1, 20). With the development of psychosomatic medicine, the ANS and HPA axis involved in stress reaction began to attract attention. Studies have found that the ANS of women with PMS was lower activated, showing inertia in activation and recovery to pressure (21, 22). Cortisol, the regulatory product of HPA axis, presented lower level under psychological stressors as well, suggesting that women with PMS endured HPA axis dysfunction (23), and the attenuation of cortisol arousal response might be a significant risk factor for PMS (24). At the same time, research on the CNS, benefited from the development of medical imaging, reported abnormal activities of brain areas involved in emotion processing among women with PMS, and suggested that the structural changes of brain tissue in amygdala, cingulate gyrus, frontal cortex and hippocampus were related to the pathogenesis of PMS (25).

It is worth noting that the above regulatory mechanisms are closely related and interact with each other. Sex hormones have known effects on neurotransmitters (26) and HPA axis (27), estrogen was reported to be related to stress susceptibility by increasing corticotropin releasing factor in central amygdala (28). The HPA axis under stress may up-regulate the expression of prolactin and unbalance the estrogen and progesterone ratio, thereby give rise to PMS (29). Additionally, the activity of ANS is link to certain brain areas. Studies reported abnormal neuromodulation in PMS group at rest (30) and inactivation of orbitofrontal cortex under acute stress (22).

At present, studies on the mechanism of PMS remain few and narrowed, and the synergistic regulation characteristics between the HPA axis, ANS and CNS have not been observed. Therefore, we conducted the comparative experiment on female college students with and without PMS, observed their HPA axis, ANS and CNS activities under anger and sadness, hoping to explore the psychological, physiological and biochemical correlations, and descript the emotional response patterns more comprehensively for PMS.

## 2. Methods

### 2.1. Participants

Two hundred twenty female college students in Beijing University of Chinese Medicine were recruited by questionnaires (including PMS scale, BAI and BDI-II), 196 students fully completed all questionnaires. In accordance with the diagnostic criteria of PMS and health, 57 students took part in the study and were divided into PMS group (*n* = 33, age = 23.36 ± 3.42 years) and healthy group (*n* = 24, age = 24.43 ± 3.06 years). There was no significant difference between the groups (Z = 1.042, *p* = 0.297).

According to John Bancroft’s PMS Scale (31) and the American College of Obstetricians and Gynecologists (ACOG), PMS was defined as:(1) scoring≥6 on PMS scale; (2) showing any one symptom of 10 somatic symptoms (breast distending pain, gastrointestinal symptoms, fatigue, headache, abdominal pain, general malaise, diet and taste changes, weight gain, sweating, skin problems) or 11 emotional symptoms (anxiety, depression, irascibility, sensitivity, easy to cry, attention deficit, reduced sociability, confusion, insomnia, increased naps, fluctuations in sexual desire) within the period of 5 days before to 3 days after the menstruation. The symptoms disappeared 4 days after the menstruation and continued to occur for 3 months.

According to World Health Organization, health was defined as: (1) scoring<45 on Beck Anxiety Inventory (BAI); (2) scoring<20 on Beck Depression Inventory (BDI); (3) qualified by physical examination, without major diseases and chronic diseases.

Participants were included into PMS group when: (a) meeting the diagnostic criteria of PMS, and (b) having regular menstrual cycles (21–35 days); and were included into healthy group when: (a) meeting the criteria of healthy, (b) having regular menstrual cycles (21–35 days), and (c) reporting no major adverse life events in the past 2 months. Individuals with psychiatric disorders were excluded.

### 2.2. Materials

#### 2.2.1. PMS scale

The scale was used for screening PMS participants and assessing the severity of PMS. The scale contains 12 items, each of which is scored in a range of 0–3 points.

#### 2.2.2. BAI

BAI contains a total of 21 items, and the scores of each item range from 1 to 4. The initial crude score should be converted into the standard score (*y* = int(1.19x)).

#### 2.2.3. BDI-II

BDI-II consists of 21 items, each of which ranged from 0 to 3 points. A total score of 0 to 13 was defined as not depressed, 14 to 19 as mild, 20 to 28 as moderate, and 29 to 63 as severe.

#### 2.2.4. Self-assessment manikin

SAM is a 9-point rating scale to assess participants’ emotional states from 3 dimensions: pleasure, arousal and dominance, representing the degree of pleasure, excitement and the sense of control. The score of each item ranges from 1 to 9 points, high score means high level of each dimension.

#### 2.2.5. Self-report inventory

Participants rated their feelings using the SRI, a 9-point rating scale with scores for each emotion ranging from 1 to 9. The inventory consists of 7 emotions (serenity, delight, anger, fear, sadness, disgust, surprise), referring to Gross (32).

### 2.3. Design and procedure

We conducted the experiment in the middle of second semester since students reported lower academic pressure in the second semester in our previous study (33). The experiment was scheduled to carry out 1–7 days before the menstruation (luteal phase) according to subjective report in the last 3 months, combined with the premenstrual discomfort of each participant. All experiments were completed between 9 and 11 a.m. Subjects were told to clean their heads before the experiment to maintain the sensitivity of electrodes. They took a break after arriving at the laboratory, wore electrode caps and fixed each electrode. After resting for 3 min, they watched angry, neutral and sad videos (the videos were presented randomly), and had a 10–15 min rest between videos. The emotional rating scales were filled out after resting and each video. The videos in our study are from the emotion video library of the State Key Laboratory of Cognitive Neuroscience and Learning at Beijing Normal University. Each video is about 3 min, scoring 5 points in emotional stimulation, with Asian leads and Chinese dubbing or subtitles.

### 2.4. Physiological and biochemical data acquisition

The physiological data was recorded by MP150 physiological multi-channel recorder of Biopac. Indicators include skin conductivity level (SCL) and heart rate variability (HRV). For HRV, we selected both time domain indices: Standard deviation of NN intervals (SDNN), root mean square of successive NN intervals differences (RMSSD), numbers of pairs of successive NN intervals that differ by more than 50 ms (NN50), and frequency domain indices: very low frequency (VLF), low frequency (LF), high frequency (HF), total power (TP) of HRV signal, LF/HF. SDNN, RMSSD, and NN50 was related to parasympathetic activity (34). TP is the total power of spectrums, reflecting the activity of ANS. VLF was reported to be closely associated to parasympathetic activity (Taylor, Carr, Myers, & Eckberg, 1998). LF was correlated with sympathetic nervous system (SNS) and part of parasympathetic nervous system (PNS). HF was linked to PNS, and LF/HF was used to describe the balance of SNS and PNS, lower LF/HF usually indicates lower sympathetic activity and higher parasympathetic activity (35). The sampling rate of physiological data was 1,000 samples/s. The sensors were attached to the pulps of left hand’s index and middle fingers, and the subjects were asked to keep their left hand as still as possible during the experiment to reduce the interference of noise on the signal. The physiological data were extracted and analyzed by AcqKnowledge 4.2, in which the standardized index of SCL (SCL = (raw-min)/(max–min)) was used because of the large basic difference between subjects.

In addition, we collected saliva samples to measure the level of salivary cortisol (SCort), and the subjects were informed not to eat irritating foods before the experiment. About 1.5 mL saliva was collected at the resting stage and 10–15 min (36) after each video, and was stored at −20°C within 2 h. The SCort were detected by Beijing Protein Innovation using competitive enzyme-linked immunosorbent assay (Salivary (Human) Cortisol ELISA SLV-2930, range: 0.09–30 ng/mL, sensitivity: 0.09 ng/mL). Meanwhile, we measured systolic blood pressure (SBP) and diastolic blood pressure (DBP) using electronic blood pressure monitor (Omron model HEM-7052) at resting stage and immediately after each video.

### 2.5. EEG data acquisition

We recorded EEG data by NuAmps 40 Channel Digital DC EEG Amplifier from Neuroscan (sampling frequency: 1000 Hz, filter bandwidth: 0-100 Hz), using bilateral mastoid as reference electrodes (A1 and A2), and 0.5 cm below the hairline of the forehead as GND. Thirty electrodes in the experiment were placed according to guidelines for standard electrode positions. Any power of electrode exceeding 100 μv was regarded as an artifact, and corrected data were exported using scan4. The EEG data was processed by MATLB with Fourier transform, filtering the power of δ band (0.15–4 Hz), θ band (4–8 Hz), α band (8–13 Hz), β band (13–25 Hz), and γ band (25–100 Hz).

### 2.6. Statistical analysis

The experiment data were analyzed using a 2 group (PMS, healthy group) × 4 condition (resting, angry, neutral, sad) repeated-measures ANOVA, and corrected according to Greenhouse–Geisser correction. The analysis mainly focused on the differences between groups. In correlation analysis, we relied on the Spearman correlation (0.1 < | *r* | < 0.3 as weak correlation, 0.3 < | *r* | < 0.5 as moderate correlation, | *r* | >0.5 as strong correlation). The threshold level for significance was *p* < 0.05. All statistical analyses were carried out in SPSS 26.0.

### 2.7. Ethics statement

This work was approved by the Ethics Committee of Beijing University of Chinese Medicine, the ethics review number is 2017BZHYLL0313. All participants take part in this study voluntarily and signed informed consent.

## 3. Results

### 3.1. Emotional self-reporting results

The emotional results showed significant condition main effects (*F*_pleasure (3,153)_ = 70.885, *p* < 0.001, η^2^ = 0.582; *F*_arousal (3,153)_ = 48.786, *p* < 0.001, η^2^ = 0.489; *F*_dominance (3,153)_ = 24.524, *p* < 0.001, η^2^ = 0.325; *F*_serenity (3,153)_ = 45.770, *p* < 0.001, η^2^ = 0.473; *F*_delight (3,153)_ = 55.825, *p* < 0.001, η^2^ = 0.523; *F*_anger (3,153)_ = 120.388, *p* < 0.001, η^2^ = 0.702; *F*_fear (3,153)_ = 3.603, *p* = 0.026, η^2^ = 0.066; *F*_sadness (3,153)_ = 79.618, *p* < 0.001, η^2^ = 0.610; *F*_disgust (3,153)_ = 62.668, *p* < 0.001, η^2^ = 0.551; *F*_surprise (3,153)_ = 3.925, *p* = 0.019, η^2^ = 0.071). No obvious group main effect and group × condition interaction effect was found. The results are shown in Table 1.

**Table 1:** The results of SAM and SRI.

	Conditions							
Resting state		Angry video		Neutral video		Sad video	
	PMS	Health	PMS	Health	PMS	Health	PMS	Health
*SAM*								
Pleasure	5.75 ± 0.219	6.143 ± 0.271	3.938 ± 0.304^∆^	3.524 ± 0.376^∆^	5.313 ± 0.213	5.381 ± 0.263	3.313 ± 0.291^∆^	2.952 ± 0.359^∆^
Arousal	2.906 ± 0.315	2.619 ± 0.389	4.813 ± 0.343^∆^	4.476 ± 0.424^∆^	3.156 ± 0.311	2.571 ± 0.383	5.000 ± 0.340^∆^	5.286 ± 0.419^∆^
Dominance	2.750 ± 0.361	2.714 ± 0.445	4.438 ± 0.329^∆^	3.952 ± 0.406^∆^	2.875 ± 0.304	2.619 ± 0.375	4.531 ± 0.379^∆^	4.381 ± 0.468^∆^
*SRI*								
Serenity	6.531 ± 0.285	7.143 ± 0.352	3.781 ± 0.347^∆^	3.810 ± 0.429^∆^	5.625 ± 0.332^*^	6.714 ± 0.410	4.031 ± 0.317^∆^	4.952 ± 0.391^∆^
Delight	5.125 ± 0.252	5.762 ± 0.311	2.719 ± 0.297^∆^	2.524 ± 0.366^∆^	4.688 ± 0.265	5.000 ± 0.328	3.063 ± 0.330^∆^	3.095 ± 0.407^∆^
Anger	1.094 ± 0.072	1.238 ± 0.089	5.219 ± 0.369^∆^	5.524 ± 0.456^∆^	1.281 ± 0.167	1.190 ± 0.207	1.688 ± 0.284	1.667 ± 0.350
Fear	1.156 ± 0.159	1.429 ± 0.197	1.500 ± 0.181	1.524 ± 0.223	1.156 ± 0.100	1.000 ± 0.123	1.625 ± 0.257	1.714 ± 0.317
Sadness	1.406 ± 0.143	1.095 ± 0.177	3.625 ± 0.445^∆^	3.810 ± 0.549^∆^	1.188 ± 0.134	1.286 ± 0.165	5.563 ± 0.390^∆^	5.667 ± 0.482^∆^
Disgust	1.281 ± 0.163	1.048 ± 0.201	4.781 ± 0.471^∆^	5.333 ± 0.581^∆^	1.156 ± 0.107	1.143 ± 0.132	2.188 ± 0.361	1.905 ± 0.446
Surprise	1.844 ± 0.283	2.143 ± 0.349	2.281 ± 0.369	2.190 ± 0.455	1.594 ± 0.208	1.381 ± 0.257	1.344 ± 0.186	1.524 ± 0.230

SAM, Self-Assessment Manikin; SRI, Self-report inventory; PMS, premenstrual syndrome. All values are mean ± standard error (SE). Using asterisk for statistic differences compared to healthy group and ^∆^ for statistic differences compared to resting state.

After watching angry video and sad video, scores in pleasure, serenity and delight significantly declined and surged in arousal, dominance, anger, sadness and disgust, among which participants scored higher in anger after angry video and higher in sadness after sad video. These results showed that the angry and sad videos successfully induced anger and sadness, respectively.

### 3.2. Physiological and biochemical results

Statistical results showed that there were significant group main effects on VLF (*F*_(1,43)_ = 4.183, *p* = 0.047, η^2^ = 0.089) and SCort (*F*_(1,48)_ = 4.709, *p* = 0.035, η^2^ = 0.089), differences were found in resting state. Significant condition main effects were observed on DBP (*F*_(3,150)_ = 8.744, *p* < 0.001, η^2^ = 0.149), SCL (*F*_(3,138)_ = 3.615, *p* = 0.015, η^2^ = 0.073), SDNN (*F*_(3,141)_ = 1435.085, *p* < 0.001, η^2^ = 0.968), RMSSD (*F*_(3,144)_ = 201.978, p < 0.001, η^2^ = 0.808), NN50 (*F*_(3,135)_ = 1050.835, *p* < 0.001, η^2^ = 0.959), VLF (*F*_(3,129)_ = 70.414, *p* < 0.001, η^2^ = 0.621), LF (*F*_(3,138)_ = 107.523, *p* < 0.001, η^2^ = 0.700), HF (*F*_(3,129)_ = 8.276, *p* < 0.001, η^2^ = 0.161), LF/HF (*F*_(3,117)_ = 70.205, *p* < 0.001, η^2^ = 0.643), TP (*F*_(3,132)_ = 53.039, *p* < 0.001, η^2^ = 0.547), SCort (*F*_(3,144)_ = 22.467, *p* < 0.001, η^2^ = 0.319).

To be specific, in PMS group, DBP significantly raised during sad video, SCL raised during both angry and sad video, and HF raised during neutral video. In both groups, SDNN, RMSSD and LF/HF dropped dramatically during sad video, VLF surged during sad video, and NN50 raised during angry video and dropped continuously during neutral and sad videos. LF raised during neutral video and declined during sad video in both groups and declined during angry video in PMS group. TP surged during sad video in both groups and declined during angry video in PMS group. SCort declined after neutral video in PMS group and declined continuously in all three video conditions in healthy group.

**Figure 1:**
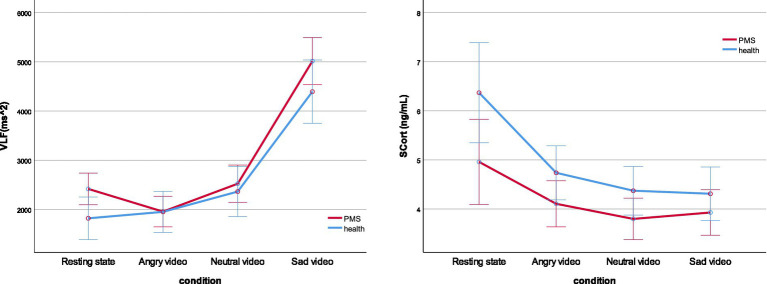
The mean values for very low frequency (VLF) and salivary cortisol (SCort). The error bars represent the 95 percent confidence intervals. (*p < 0.05).

No significant interaction effect was found. The VLF and SCort results are shown in Figure 1, and the mean value of each parameter in different conditions and groups are shown in Table 2.

**Table 2:** The physiological and biochemical indices results.

	Conditions							
Resting state		Angry video		Neutral video		Sad video	
	PMS	Health	PMS	Health	PMS	Health	PMS	Health
SBP	101.656 ± 1.156	101.8 ± 1.462	100.094 ± 1.349	98.7 ± 1.707	100.469 ± 1.398	99.15 ± 1.768	102.188 ± 1.356^∆^	99.25 ± 1.715
DBP	61.625 ± 1.107	60.7 ± 1.401	61.125 ± 0.971	59.5 ± 1.228	60.469 ± 0.941	59.75 ± 1.191	64.5 ± 1.077	62.15 ± 1.362
SCL	0.313 ± 0.019	0.327 ± 0.023	0.381 ± 0.022^∆^	0.372 ± 0.027	0.374 ± 0.02	0.336 ± 0.024	0.4 ± 0.024^∆^	0.333 ± 0.029
SDNN	0.469 ± 0.008	0.449 ± 0.01	0.463 ± 0.008	0.461 ± 0.01	0.462 ± 0.008	0.444 ± 0.01	0.134 ± 0.004^∆^	0.134 ± 0.005^∆^
RMSSD	0.318 ± 0.008	0.309 ± 0.01	0.317 ± 0.007	0.313 ± 0.009	0.312 ± 0.008	0.307 ± 0.011	0.2 ± 0.006^∆^	0.2 ± 0.008^∆^
NN50	248.379 ± 5.251	242.667 ± 6.665	271 ± 5.105^∆^	271.278 ± 6.48^∆^	190.483 ± 3.652^∆^	180.556 ± 4.636^∆^	55.897 ± 3.373^∆^	59.167 ± 4.281^∆^
VLF	2417.728 ± 159.469^*^	1820.739 ± 214.691	1956.706 ± 153.832	1952.251 ± 207.102	2524.357 ± 187.401	2364.175 ± 252.296	5015.111 ± 236.662^∆^	4395.189 ± 318.617^∆^
LF	1026.418 ± 42.236	994.735 ± 54.526	914.621 ± 48.135^∆^	871.608 ± 62.142	1299.07 ± 65.641^∆^	1204.876 ± 84.742^∆^	526.547 ± 26.398^∆^	570.403 ± 34.08^∆^
HF	191.805 ± 12.734	162.788 ± 17.144	172.487 ± 13.088	156.142 ± 17.62	233.493 ± 18.481^∆^	200.303 ± 24.881	197.392 ± 9.766	196.951 ± 13.148
LF/HF	5.446 ± 0.207	5.906 ± 0.272	5.648 ± 0.284	5.452 ± 0.373	5.822 ± 0.356	6.241 ± 0.468	2.679 ± 0.112^∆^	2.919 ± 0.148^∆^
TP	3664.491 ± 179.676	2906.243 ± 234.673	3006.247 ± 192.5^∆^	2933.566 ± 251.424	4059.785 ± 207.218	3577.728 ± 270.647	5741.208 ± 278.832^∆^	5507.753 ± 364.181^∆^
Scort	4.959 ± 0.431^*^	6.368 ± 0.507	4.105 ± 0.233	4.737 ± 0.274^∆^	3.797 ± 0.210^∆^	4.372 ± 0.247^∆^	3.928 ± 0.23	4.31 ± 0.271^∆^

PMS, premenstrual syndrome; SCL, skin conductivity level; SDNN, standard deviation of NN intervals; RMSSD, root mean square of successive NN intervals differences; NN50, numbers of pairs of successive NN intervals that differ by more than 50 ms; VLF, very low frequency; LF, low frequency; HF, high frequency; TP, total power of HRV signal; SCort, salivary cortisol. All values are mean ± standard error (SE). Using asterisk for statistic differences compared to healthy group and ^∆^ for statistic differences compared to resting state.

### 3.3. EEG results

There was significant condition main effect on grand mean of θ band (*F*_(3,144)_ = 4.776, *p* = 0.003, η^2^ = 0.090), α band (*F*_(3,144)_ = 36.851, *p* < 0.001, η^2^ = 0.434), γ band (*F*_(3,144)_ = 9.279, *p* < 0.001, η^2^ = 0.162), and interaction effect on α band (*F*_(3,72)_ = 3.382, *p* = 0.040, η^2^ = 0.066) and γ band (*F*_(3,72)_ = 3.330, *p* = 0.021, η^2^ = 0.065). No group main effect was found. Simple effect analysis showed that the α band power of PMS group (*F*_(3,72)_ = 40.04, *p* < 0.001, η^2^ = 0.440) and healthy group (*F*_(3,72)_ = 10.00, *p* < 0.001, η^2^ = 0.164) during sad video were lower than that during the other three conditions. For γ band, the power of PMS group (*F*_(3,72)_ = 6.61, *p* < 0.001, η^2^ = 0.115) during angry video was higher than that during neutral and sad video, and the power of healthy group (*F*_(3,72)_ = 4.85, *p* = 0.003, η^2^ = 0.087) during sad video was lower than that during resting state.

Among specific electrodes, there was significant group main effect on β band power at T_6_ (*F*_(1,51)_ = 4.235, *p* = 0.045, η^2^ = 0.077), and interaction effects on δ band power at F_7_ (*F*_(3,78)_ = 5.268, *p* = 0.004, η^2^ = 0.094), *F*_8_ (*F*_(3,78)_ = 4.404, *p* = 0.017, η^2^ = 0.079), α band power at CP_3_ (*F*_(3,78)_ = 4.177, *p* = 0.032 η^2^ = 0.076), CP_4_ (*F*_(3,78)_ = 3.696, *p* = 0.041, η^2^ = 0.068), TP_8_ (*F*_(3,78)_ = 2.725, *p* = 0.046, η^2^ = 0.051), T_5_ (*F*_(3,78)_ = 3.527, *p* = 0.016, η^2^ = 0.065), P_3_ (*F*_(3,78)_ = 4.533, *p* = 0.023, η^2^ = 0.082), P_4_ (*F*_(3,78)_ = 3.890, *p* = 0.033, η^2^ = 0.071), and γ band power at F_3_ (*F*_(3,78)_ = 3.358, *p* = 0.020, η^2^ = 0.062), F_Z_ (*F*_(3,78)_ = 2.721, p = 0.046, η^2^ = 0.051) FC_3_ (F_(3,78)_ = 3.092, *p* = 0.029, η^2^ = 0.057).

**Figure 2:**
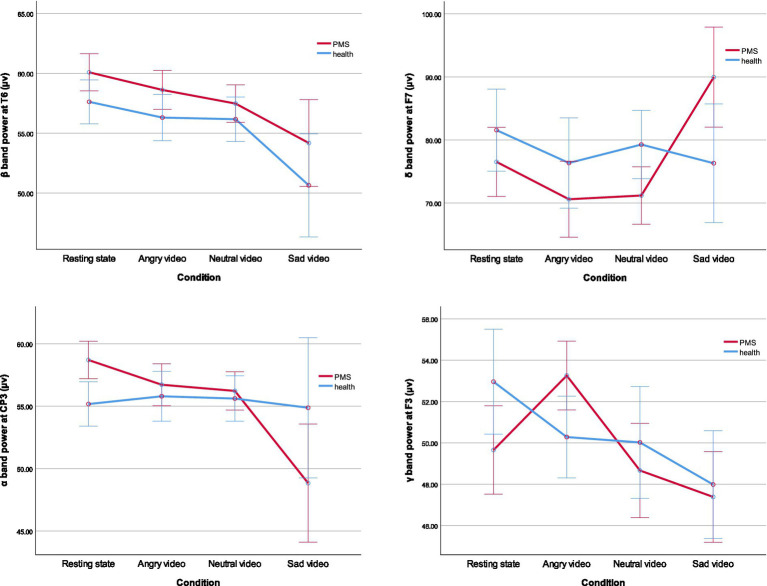
The mean value of β band power at T6, δ band power at F7, α band power at CP3, and γ band power at F3. The error bars represent the 95 percent confidence intervals. (*p < 0.05).

Simple effect analysis showed that the δ band power at F_7_ and F_8_ of PMS group was lower than that of healthy group during neutral video (*F*_delta F7 (1,51)_ = 5.261, *p* = 0.026, η^2^ = 0.094; *F*_delta F8(1,51)_ = 4.076, *p* = 0.049, η^2^ = 0.074), but higher than that of healthy group during sad video (*F*_delta F7 (1,51)_ = 4.968, *p* = 0.030, η^2^ = 0.089; *F*_delta F8(1,51)_ = 5.112, *p* = 0.028, η^2^ = 0.091). The α band power at CP_3_, CP_4_, P_3_ and P_4_ of PMS group was higher than that of healthy group at rest (*F*_alpha CP3 (1,51)_ = 6.119, *p* = 0.017, η^2^ = 0.107; *F*_alpha CP4(1,51)_ = 4.347, *p* = 0.042, η^2^ = 0.079; *F*_alpha P3 (1,51)_ = 9.288, *p* = 0.004, η^2^ = 0.154; *F*_alpha P4(1,51)_ = 5.399, *p* = 0.024, η^2^ = 0.096). The γ band power at F_3_, F_Z_, and FC_3_ of PMS group was higher than that of healthy group during angry video (*F*_gamma F3 (1,51)_ = 5.338, *p* = 0.025, η^2^ = 0.095; *F*_gamma FZ(1,51)_ = 5.022, *p* = 0.029, η^2^ = 0.090; F_gamma FC3 (1,51)_ = 5.016, *p* = 0.029, η^2^ = 0.090). The main results are shown in Table 3, and outcomes of β band power at T_6_, δ band power at F_7_, α band power at CP_3_ and γ band power at F_3_ are shown in Figure 2.

**Table 3:** Main results on EEG.

	Conditions							
Resting state		Angry video		Neutral video		Sad video	
	PMS	Health	PMS	Health	PMS	Health	PMS	Health
alpha	57.751 ± 0.561	56.246 ± 0.687	57.037 ± 0.586	56.449 ± 0.717	55.674 ± 0.543^∆^	55.553 ± 0.665	48.364 ± 1.236^∆^	51.481 ± 1.514^∆^
gamma	50.124 ± 0.668	51.721 ± 0.818	51.809 ± 0.657	50.052 ± 0.805	49.087 ± 0.615	49.931 ± 0.753	48.665 ± 0.763	51.481 ± 1.515^∆^
beta T6	60.087 ± 0.771	57.621 ± 0.915	58.618 ± 0.809	56.308 ± 0.96	57.475 ± 0.78	56.168 ± 0.926	54.18 ± 1.807	51.481 ± 1.516^∆^
delta F7	76.526 ± 2.731	81.563 ± 3.242	70.599 ± 3.004	76.348 ± 3.565	71.185 ± 2.272^*∆^	79.274 ± 2.697	89.969 ± 3.949^*^	51.481 ± 1.517^∆^
delta F8	79.325 ± 3.052	80.356 ± 3.622	70.27 ± 3.382	75.275 ± 4.014	72.13 ± 2.438^*∆^	79.77 ± 2.894	104.396 ± 5.454^*^	51.481 ± 1.518^∆^
alpha CP3	58.432 ± 0.701^*^	55.739 ± 0.833	55.781 ± 0.917	55.909 ± 1.088	55.52 ± 0.812	54.774 ± 0.964	48.977 ± 2.873^∆^	51.481 ± 1.519^∆^
alpha CP4	58.68 ± 0.739^*^	56.287 ± 0.878	57.29 ± 0.575	56.481 ± 0.682	55.231 ± 0.855^∆^	54.916 ± 1.015	50.112 ± 2.386^∆^	51.481 ± 1.520^∆^
alpha TP8	56.714 ± 0.871	54.027 ± 1.034	55.339 ± 0.905	54.645 ± 1.074	53.567 ± 0.923	52.603 ± 1.095	45.633 ± 0.871^∆^	51.481 ± 1.521^∆^
alpha T5	57.342 ± 0.96	54.74 ± 1.139	55.834 ± 0.89	54.994 ± 1.057	54.698 ± 0.9	54.606 ± 1.069	45.983 ± 0.997^∆^	51.481 ± 1.522^∆^
alpha P3	58.701 ± 0.746^*^	55.172 ± 0.886	56.72 ± 0.837	55.795 ± 0.994	56.223 ± 0.764	55.613 ± 0.906	48.838 ± 2.357^∆^	51.481 ± 1.523^∆^
alpha P4	59.068 ± 0.761^*^	56.323 ± 0.903	56.791 ± 0.756	56.614 ± 0.897	56.872 ± 0.728	55.548 ± 0.864	49.552 ± 2.231^∆^	51.481 ± 1.524^∆^
gamma F3	49.655 ± 1.064	52.96 ± 1.264	53.257 ± 0.83^*∆^	50.282 ± 0.985	48.664 ± 1.136	50.022 ± 1.348	47.384 ± 1.092	51.481 ± 1.525^∆^
gamma FZ	49.482 ± 0.858	51.108 ± 1.019	51.652 ± 0.781^*^	48.937 ± 0.927	47.969 ± 1.013	48.607 ± 1.202	44.798 ± 0.537^∆^	51.481 ± 1.526^∆^
gamma FC3	48.874 ± 1.001	51.779 ± 1.188	51.927 ± 0.843^*^	48.996 ± 1.001	47.512 ± 1.059	48.468 ± 1.257	46.029 ± 1.168	51.481 ± 1.527^∆^

Alpha, grand mean of alpha band power; gamma, grand mean of gamma band power. All values are mean ± standard error (SE). Using asterisk for statistic differences compared to healthy group and ^∆^ for statistic differences compared to resting state.

### 3.4. Correlation analysis

We used spearman correlation to analyze the relations between the score of PMS scale and the variations of indices in different video stages (compared with the resting stage, denoted by Δ below). The results showed that, among PMS participants, the score of PMS scale was positively correlated with Δθ band power (*r* = 0.434, *p* = 0.015) during sad video, negatively correlated with ΔSBP (*r* = −0.362, *p* = 0.039) and ΔScort (*r* = −0.529, *p* = 0.002) during angry video and ΔScort (*r* = −0.379, *p* = 0.036) during neutral video. The correlations between the score of PMS scale and the EEG data are shown in Table 4.

**Table 4:** The correlations between the score of PMS scale and the EEG data of specific electrodes in 3 video stages.

Angry video			Neutral video			Sad video		
Indices	*r*	*p*	Indices	*r*	*p*	Indices	*r*	*p*
Δtheta FC_4_	0.413^*^	0.021	Δtheta C_4_	0.390^*^	0.030	Δtheta F_4_	0.358^*^	0.048
Δtheta C_4_	0.501^**^	0.004	Δtheta P_Z_	0.418^*^	0.019	Δtheta FC_4_	0.360^*^	0.047
Δtheta CP_3_	0.447^*^	0.012	Δalpha T_6_	−0.411^*^	0.022	Δtheta C_4_	0.432^*^	0.015
Δalpha F_7_	0.381^*^	0.034	Δalpha O_1_	−0.376^*^	0.037	Δtheta CP_Z_	0.462^**^	0.009
Δalpha F_Z_	0.382^*^	0.034	Δbeta T_3_	−0.379^*^	0.036	Δtheta P_Z_	0.518^**^	0.003
Δalpha FC_Z_	0.437^*^	0.014	Δgamma FP_1_	0.363^*^	0.045			
Δalpha C_Z_	0.373^*^	0.039	Δgamma F_3_	0.369^*^	0.041			
Δgamma F_Z_	0.368^*^	0.042	Δgamma F_Z_	0.598^***^	0.000			
			Δgamma F_4_	0.386^*^	0.032			
			Δgamma FC_3_	0.387^*^	0.032			
			Δgamma FC_Z_	0.543^**^	0.002			
			Δgamma FC_4_	0.360^*^	0.046			
			Δgamma C_Z_	0.377^*^	0.037			

*p < 0.05, **p < 0.01, ***p < 0.001.

**Figure 3:**
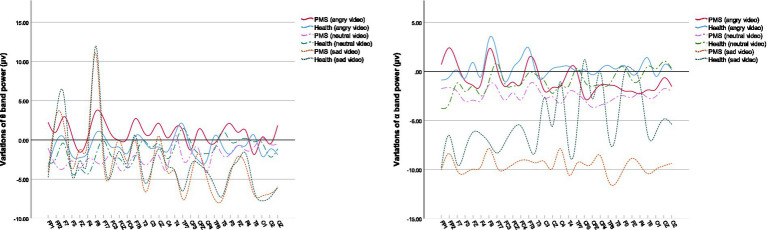
The variations of θ and α band power in 3 video conditions.

Among the power at specific electrodes, the fluctuations of θ band and α band were typically associated with PMS. Their variations are shown in Figure 3.

## 4. Discussion

In our study, we observed no statistic difference for PMS participants compared to healthy group in self-report emotions as well as HPA axis and ANS activities during each video stage. However, PMS participants showed distinct lower activity of HPA axis and higher activity of PNS in resting state. Besides, the reduced HPA and SNS activities during angry video (compared with the resting stage) were moderately related to higher PMS score. An inverse relation between the HPA reactivity and PMS during neutral video was also observed. Additionally, the results implied that the HPA axis responded strongly to anger, while ANS is more sensitive to sadness. As to EEG results, compared to healthy group, PMS participants showed different response patterns in α and β bands at rest, in which the higher inhibition in parietal lobe and arousal in right posterior temporal lobe were remarkable. Intense arousal of γ in the left frontal lobe during angry video, reduced arousal of δ in anterior temporal lobe during neutral video as well as increased arousal of δ in anterior temporal lobe were also observed among PMS participants.

### 4.1. Psychological characteristics

PMS participants reported lower scores on serenity for neutral video. This result suggested that for students with PMS, neutral stressors also stirred their emotions. Another explanation would be the persistent effect of other negative emotional videos. Previous studies implied that women with PMS might suffer from a weakening of positive experience (37), and even had trouble disengaging attention from negative emotions in severe cases (38), which is consistent with our results. However, since there was no significant group main effect, study with a larger sample size is necessary to get a definite conclusion.

### 4.2. The activities of the HPA axis

Salivary cortisol, as an indicator of HPA axis’ activity, is an important regulatory hormone of stress reaction (39). Both acute and chronic stress reactions activate the HPA axis, leading to an increase in cortisol (40–42). The cortisol content reaches its peak after 10 min in acute stress (36). The low level and slow response of cortisol weakened the reaction capacity to psychological stress (43). The results that the salivary cortisol content of PMS group at rest was significantly lower than that of the healthy group, might indicate a weaker activity of the HPA axis for college students with PMS, which impaired psychological resilience and brought about more negative feelings (44). Other studies reported blunted cortisol reactivity to Trier Social Stress Test in women with PMS (45) and lower cortisol levels at rest and under mental stress in women with premenstrual dysphoric disorder (PMDD) (46). In our study, no statistic difference between the groups could prove a distinct response pattern of HPA axis in PMS participants to emotional videos, but the correlation analysis indicated an inverse relationship between the activity of HPA axis and PMS symptoms in coping with angry and neutral stressors.

It is notable that cortisol decreased significantly in video conditions compared to resting state, which appears to contradict general cognition and assumptions (47), but is consistent to some earlier research (48, 49). As participants seemed relatively stable at rest according to *post hoc* multiple comparison of mood scores, we excluded the stress caused by experiment itself, which might give rise to a high level of cortisol at baseline, and speculated that our emotion induction inhibited the HPA axis’ activity.

Furthermore, *post hoc* multiple comparison showed that the cortisol level after viewing angry video was higher than that after sad video, and was remarkably higher than that after neutral video. No statistical difference between the latter two. This indicated a higher activity of HPA axis in anger compared with sadness.

### 4.3. The activities of ANS

HRV is a non-invasive indicator for ANS activity. Our study found that compared with healthy participants, PMS participants showed higher VLF at rest. Considered all indicators, our findings more likely indicated stronger parasympathetic activities among PMS students in luteal phase, which was inconsistent with previous research (50, 51). Since there is no accordant conclusion about the relationship between VLF and PNS, the explanation needs to be taken with caution.

Under the stimulation of sadness, among all the participants, the VLF and TP increased significantly, and no difference was observed in HF compared with the baseline. The other indexes showed a downward trend. It is interesting that LF and LF/HF declined significantly, which mainly represented the weakening of sympathetic activity or the strengthening of parasympathetic activity, yet SDNN, RMSSD and NN50 also drastically reduced, suggesting a decreased activity of PNS. Contrary to common believes, researches questioned the utility of LF, HF and LF/HF as indicators in measuring sympathetic and parasympathetic activities because the physiological basis of them are hard to be confirmed (52, 53). These contradictory outcomes might implicitly approve the inaccuracy of LF and LF/HF. Together, we prefer to believe that the sympathetic activity strengthened and parasympathetic activity got weakened, and the overall activity of ANS, which was less sensitive to anger, was notably enhanced after the sad video. Similar result has been reported (54). The study claimed that lower parasympathetic activity may interfere self-regulation and behavioral flexibility. We therefore believe that the sad video in this experiment triggered stronger negative affectivity.

Additionally, the changes of SBP after inducing anger, compared to the baseline, correlated negatively and moderately with the degree of PMS. SBP is associated with the sympathetic activity (55). Research claimed that, compared with control subjects, women with PMS showed higher SBP and DBP at baseline and after cold pressor test (56), which seems to contradict our findings. However, reduced SBP and DBP were reported to be related to depression, anxiety and stress (57, 58). The difference in results may partly come from the different stressors.

### 4.4. Interaction between HPA axis and ANS

Taken together with the results above, angry video did not seem to result in detectable stress response, and even inhibited the activity of HPA axis and SNS. Another explanation is that long-term pressure has changed the response pattern of the HPA axis, since chronic psychological stress substantially works on the HPA axis (59), and the sustained high activation may wear out the resilience, thus impairs acute stress response (60). In contrast, sadness elicited more intense response in ANS, which means stronger emotional perception. For ANS, previous research reported lower specificity in response to stress stimuli and poor correlation with PMS, compared with HPA axis (45). However, our results suggested that the activities of both HPA axis and ANS had certain bearing on PMS, especially in response to anger.

### 4.5. The activities of CNS regulation

PMS participants presented different patterns of CNS activities under all four conditions in different bands, prominently in δ, α and γ bands.

In the resting state, higher power of β band in the right posterior temporal lobe and α band in the parietal lobe were observed in PMS group. α frequency band mainly suppresses the cortex, manifesting as enhanced power and increased inhibition under the stimulation of negative emotions, which thereupon narrows attention and range of cognitive (61). Liu et al. (10) also reported higher α activity in women with PMS at rest. β band is associated with maintaining the current sensorimotor or cognitive state (62) and is enhanced to keep attention when in high arousal of negative emotions (63). The result indicated that, for PMS students in resting state, heightened parietal inhibition and right posterior temporal arousal might be allied to the low activity of HPA axis and the high activity of PNS. However, previous studies mainly reported heightened activities in the left temporal lobe among PMS participants (64, 65). We suspected that the different recording time could be responsible for part of the inconformity.

During the angry video, PMS group manifested higher power of γ band in the left frontal lobe, and the variation of γ band power in frontal midline was positively related to the severity of PMS. γ band is involved in visual processing and sensitive to emotional stimuli (66, 67). Its enhancement is positively correlated with subjective emotional arousal (68), and is linked to sustained emotional elaboration (69). In addition, the orbitofrontal cortex (OFC) is essential in emotion processing, in which γ band will be activated when dealing with negative emotions and feedback (70).

Besides, in angry phase, α band power mainly heightened in healthy group, which is accord with the conclusion that negative emotions raise α band power, while that in PMS group mainly declined. However, in left anterior temporal lobe and frontal midline, the variation of α band power was positively correlated with the score of PMS, that is, the manifestation of PMS might get worse with higher inhibitory effect of α band in mentioned lobe when dealing with anger.

During neutral video, the δ band power of the anterior temporal lobe in PMS group was lower than that in healthy group. The results during the sad video were in contrast. δ band plays a major role in the cognitive process. It was reported to be more responsive to emotional stimulation, especially to negative emotions, and sensitive participants showed larger increments in δ band (63). Our results suggested that the δ band of students with PMS had relatively poor response to neutral stimulation but significantly stronger response to sadness, indicating a susceptibility to sad emotion in the anterior temporal lobe. Previous study also claimed that people with PMDD had higher activation in the temporal lobe under emotional stimulation (19).

We found it interesting that the increased γ band power in frontal lobe during neutral video was also in line with a higher PMS score. Research reported more intensive high γ band (50–80 Hz) to negative stimuli (71). Our results suggested that, among PMS participants, γ band correlated moderately with anger as well as neutral stressors in frontal lobe, especially in frontal midline, which might have some relation to reduced SCort. Moreover, declined power of α band in right posterior temporal and left occipital lobes, and β band in left middle temporal lobe was moderately associated with worse PMS manifestation.

During sad video, the PMS score was positively related to the variation of θ band power, prominently in the right frontal lobe, right central sulcus and parietal midline. Similarly, the increased power of θ band in right frontal lobe, right central sulcus and left parietal lobe during angry video, in the right central sulcus and parietal midline during neutral video was also positively correlated with PMS scores. θ activity was reported to be associated with cognitive demand (72). Higher θ activity in central and parietal lobe, similar regions in our correlation results, was observed among major depressive disorder (73). The results possibly suggested that PMS participants with more severe symptoms engaged more attention on videos.

In conclusion, our study implied that subjects with PMS had lower SCort and higher VLF at baseline, possibly indicating their lower activity of HPA axis and higher activity of PNS, which might be associated with the heightened inhibition of α band on the parietal lobe and the increased arousal of β band on the right posterior temporal lobe. In emotional regulation, the severity of PMS was moderately related to the decrease of Scort and the enhancement of γ in frontal midline during angry and neutral videos. The enhanced θ in right central sulcus and parietal lobe during all three conditions, the increased inhibition of α in left anterior temporal lobe and frontal midline under anger, the weakened inhibition of α in right posterior temporal and left occipital lobes as well as weakened β activity in left middle temporal lobe under neutral video also had medium correlations with more severe PMS. We speculate that γ activity in frontal lobe may play a role in lower SCort in dealing with angry and neutral stressors, but in the case of dealing with sadness, no distinct response pattern had been found in the HPA axis and ANS, different from the CNS results. It cannot be dismissed that, no intergroup differences were observed in subjective emotion scores for either sadness or anger, and the result did not correspond to physiological manifestation. We suspect that the conflict might stem from the suppression use that contributes to hide feelings (32, 74), yet more research is needed to be sure.

## Data availability statement

The raw data supporting the conclusions of this article will be made available by the authors, without undue reservation.

## Ethics statement

The studies involving humans were approved by Ethics Committee of Beijing University of Chinese Medicine. The studies were conducted in accordance with the local legislation and institutional requirements. The participants provided their written informed consent to participate in this study.

## Author contributions

YZ, XH, and JX contributed the study structure. HW, XW, and YW collected the experimental data. JX, SY, HW, and XW conducted the data analysis. JX wrote the first draft of the manuscript. YZ and XH made critical revisions. All authors approved the submitted version of this manuscript.

## Funding

This work was supported by the National Natural Science Foundation of China (General Program) (No. 81373771).

## Conflict of interest

XW was employed by Chemical Industry Press Co., LTD.

The remaining authors declare that the research was conducted in the absence of any commercial or financial relationships that could be construed as a potential conflict of interest.

## Publisher’s note

All claims expressed in this article are solely those of the authors and do not necessarily represent those of their affiliated organizations, or those of the publisher, the editors and the reviewers. Any product that may be evaluated in this article, or claim that may be made by its manufacturer, is not guaranteed or endorsed by the publisher.
