# Supplementary material for: Psychological, physiological, and biochemical correlations after negative emotional videos in college students with and without premenstrual syndrome
Source: Front Psychiatry. 2023 Aug 15;14:1228276. doi: 10.3389/fpsyt.2023.1228276 (PMC10463732; doi:10.3389/fpsyt.2023.1228276)
Supplement: Supplementary file 1 [file Data_Sheet_1.docx]

Supplementary Material

**Negative emotion regulation in premenstrual syndrome: a comprehensive study of central nervous system, ANS and HPA axis**

**Jingyu Xing, Hao Wu, Xue Wang, Shuang Yi, Yu Wei, Yan Zhao^*^, Xingang Hu^*^**

*** Correspondence:** Yan Zhao: [yanzh3232@126.com](mailto:yanzh3232@126.com); Xingang Hu: [xingganghu@163.com](mailto:xingganghu@163.com)

## 1 Supplementary Figures


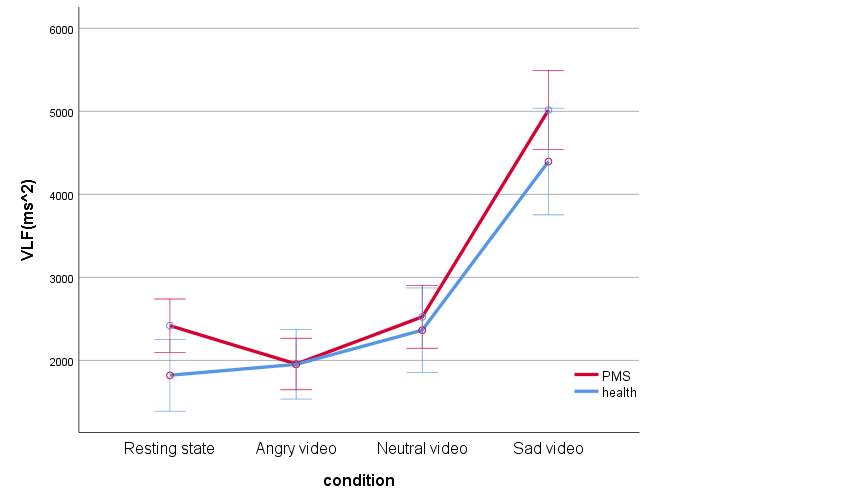

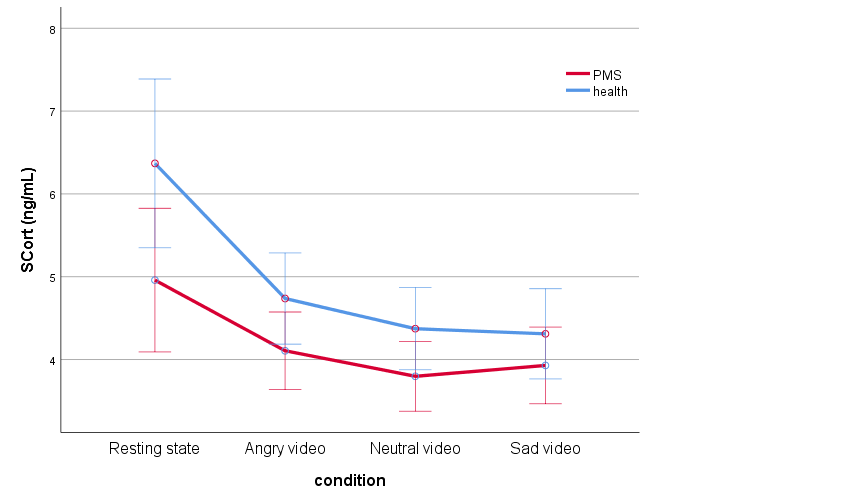


*

*

**Supplementary Figure 1.** The mean values for very low frequency (VLF) and salivary cortisol (SCort). The error bars represent the 95 percent confidence intervals. (* p<.05).


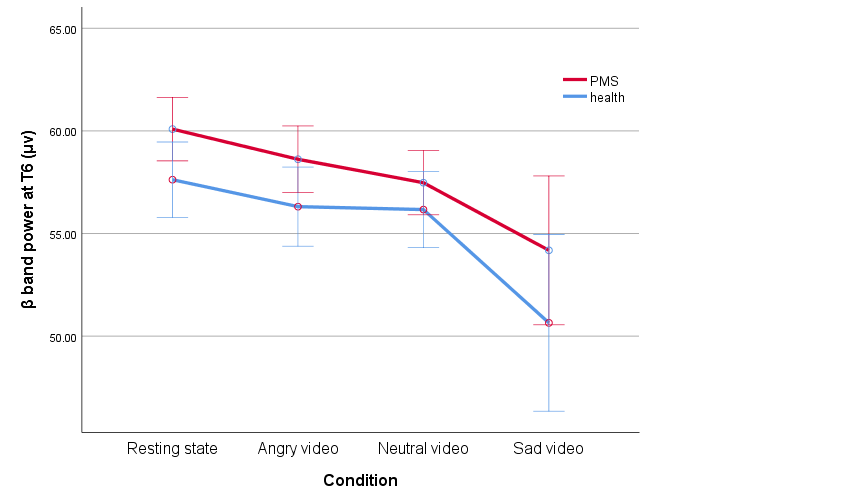


*


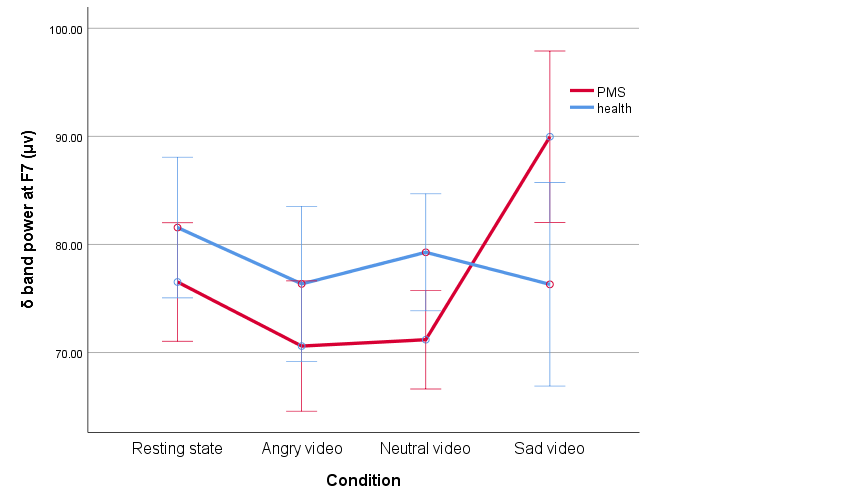


*

*


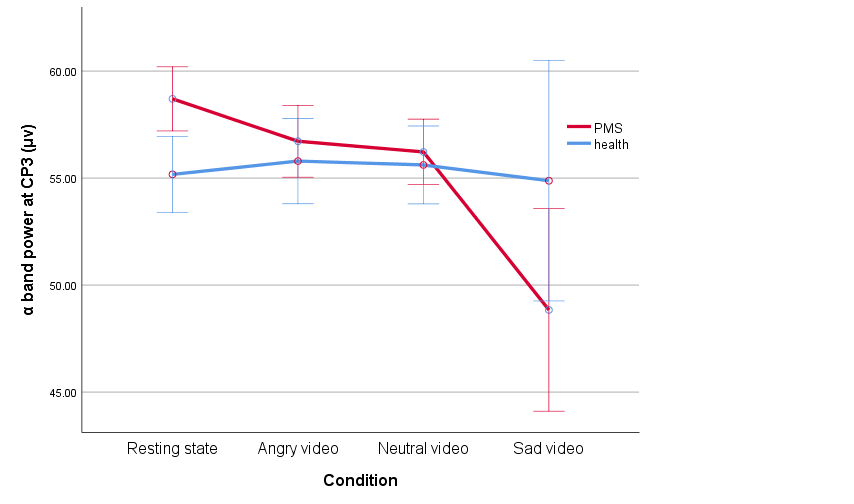


*


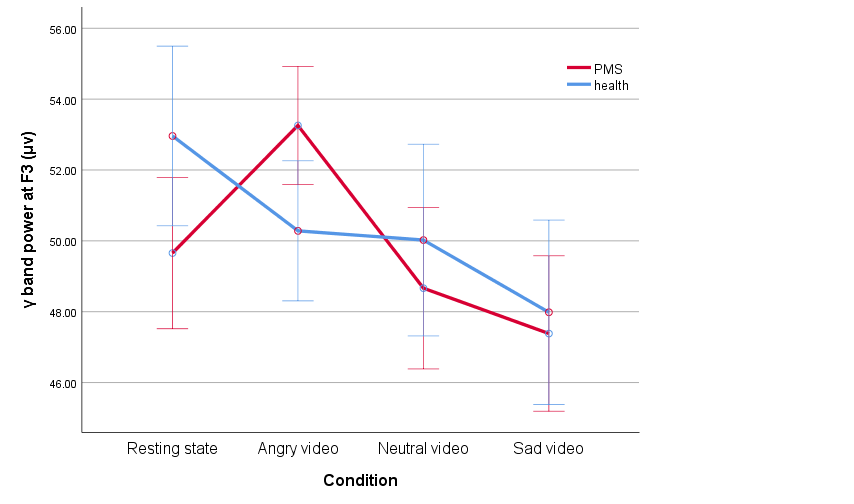


*

**Supplementary Figure 2.** The mean value of β band power at T6, δ band power at F7, α band power at CP3 and γ band power at F3. The error bars represent the 95 percent confidence intervals. (* p<.05).


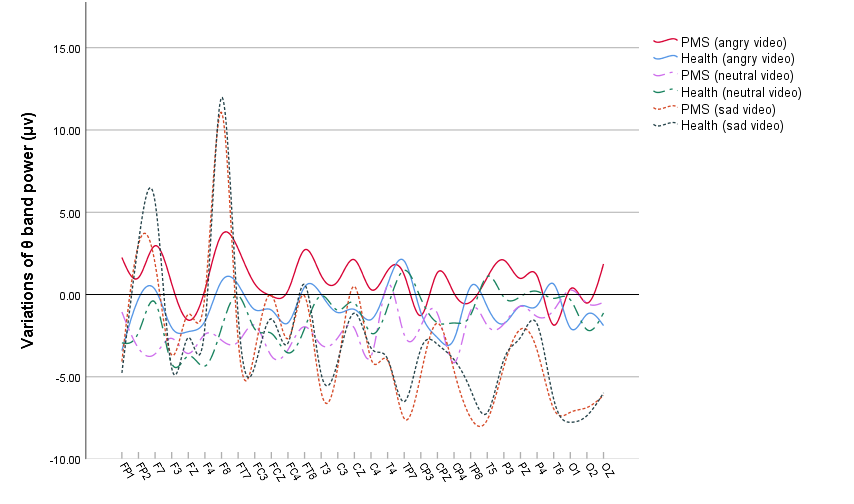

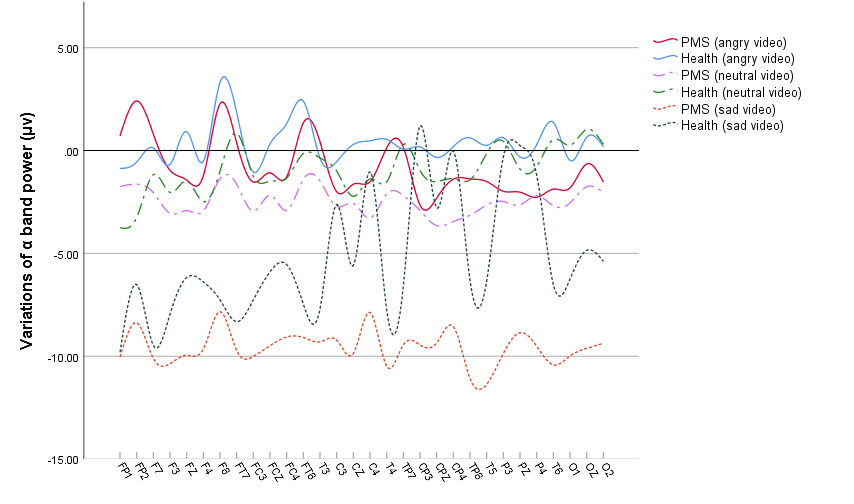


**Supplementary Figure 3.** The variations of θ and α band power in 3 video conditions.
